# Bioinformatic analysis, clinical implications and experimental validation of ferroptosis-related feature gene in IgA nephropathy: focus on DUSP1

**DOI:** 10.3389/fmed.2025.1612200

**Published:** 2025-08-07

**Authors:** Tingting Liu, Tingting Pan, Mingxin Chang, Shaojie Fu, Hongzhao Xu, Hao Wu, Zhonggao Xu, Yanli Cheng

**Affiliations:** ^1^Department of Nephrology, The First Hospital of Jilin University, Changchun, China; ^2^Department of Anaesthesiology, The First Hospital of Jilin University, Changchun, China; ^3^Nephrology Department, The Affiliated Hospital to Changchun University of Chinese Medicine, Changchun, Jilin, China

**Keywords:** IgA nephropathy, ferroptosis, machine learning, DUSP1, immune cell infiltration

## Abstract

**Background:**

Immunoglobulin A nephropathy (IgAN), recognized as the leading cause of primary glomerular disease worldwide, continues to present unresolved complexities in its underlying pathogenic mechanisms. Emerging evidence underscores ferroptosis, an iron-mediated regulated cell death pathway driven by the accumulation of lipid peroxides, as a potential contributor to various pathological conditions. Despite growing interest in this field, the exact molecular pathways governing ferroptosis activation in IgAN progression remain incompletely understood and require systematic investigation. The aim of this study was to identify ferroptosis-related feature gene (FFG) for the potential diagnosis of IgAN and to investigate its relationship with renal immune cell infiltration.

**Methods:**

Renal tissue microarray datasets (GSE93798, GSE104948, GSE99339) from IgAN patients and normal controls were retrieved from GEO database. The ferroptosis-related genes were obtained from the Ferrb database. Machine learning algorithms (LASSO, SVM-RFE, random forest) were employed to screen FFGs. The findings were validated in an IgAN mouse model using immunohistochemistry and western blotting. Gene set enrichment analysis (GSEA) was conducted to explore the underlying mechanism of FFG in IgAN. Immune cell infiltration characteristics were also analyzed vis CIBERSORT algorithm.

**Results:**

A total of 180 ferroptosis-related differentially expressed genes were identified in IgAN. Among them, dual specificity phosphatase 1 (DUSP1) was screened as FFG by three machine learning algorithms. DUSP1 exhibited significant downregulation in renal tissues of both IgAN patients and mice. Enhanced transcriptional abundance demonstrated significant positive associations with ferroptosis-associated biomarkers glutathione peroxidase-4 (GPX4) and cystine/glutamate antiporter (SLC7A11/xCT), while displaying an inverse relationship with acyl-CoA synthetase long-chain isoform 4 (ACSL4) expression. GSEA further identified DUSP1’s functional enrichment in critical signaling networks, particularly mitogen-activated protein kinase (MAPK) cascades, ERBB receptor tyrosine kinase pathways, and Janus kinase-signal transducer (JAK–STAT) transduction mechanisms. Immunoinfiltration analysis demonstrated increased infiltration of T follicular helper cells, activated NK cells, and M1 macrophages in the renal tissues of IgAN patients, with DUSP1 expression showing negative correlations with these proinflammatory cell types.

**Conclusion:**

Our research successfully identified DUSP1 as a ferroptosis-related biomarker in IgAN patients, and explored its potential mechanism in the pathogenesis of IgAN and its potential relationship with immune cell infiltration. These findings are of great significance for the diagnosis and prospective treatment strategies for IgAN patients.

## 1 Introduction

Immunoglobulin A nephropathy (IgAN), clinically designated as Berger’s disease (1, 2), constitutes the predominant subtype of primary glomerular nephropathy worldwide (3). Its hallmark pathological feature involves immune complex deposition in the glomerular mesangial region, with IgA as the core component, and may include concurrent C3 deposition. Clinically, IgAN presents with heterogeneous manifestations, ranging from asymptomatic microscopic hematuria and proteinuria to acute hypertension or nephrotic syndrome (4). The exact pathogenesis of IgAN remains incompletely understood but is believed to involve the interplay of genetic predisposition, mucosal immune dysregulation, and aberrant glycosylation of IgA1 molecules (5). The absence of validated diagnostic biomarkers and precise therapeutic interventions contributes to delayed disease recognition and heterogeneous clinical trajectories across affected populations. Epidemiological data indicate that 30–40% of cases advance to end-stage renal disease (ESRD) within a 20-year post-diagnosis interval, establishing this condition as a predominant contributor to renal replacement therapy requirements in individuals aged 20–50 years (6). This progression imposes a substantial burden on the healthcare system. Consequently, elucidating the fundamental mechanisms underlying IgAN and identifying key regulatory genes are critical priorities for enhancing diagnostic accuracy, therapeutic efficacy, and the development of precision medicine approaches.

Ferroptosis constitutes an iron-mediated regulated cell death modality defined mechanistically by the uncontrolled oxidation of polyunsaturated fatty acids within cellular membranes. This molecular mechanism differs significantly from apoptosis, necrosis, autophagy, and other modes of cell death (7, 8). The core pathological process involves the Fenton reaction induced by iron overload. This process culminates in the dysregulated generation of oxygen-derived free radicals (reactive oxygen species, ROS) and consequent breakdown of cellular antioxidant defense systems. These factors drive the lipid peroxidation chain reaction, ultimately causing the collapse of the cellular membrane system (9). While ferroptosis has been implicated in various renal pathologies including acute kidney injury, renal fibrosis, and diabetic nephropathy (4, 10–12), its role in IgAN remains incompletely understood. Notably, clinical observations suggest a plausible link between iron metabolism and IgAN progression. Elevated serum ferritin and transferrin saturation, markers of systemic iron dysregulation, correlate with tubular interstitial damage and poorer prognosis in IgAN patient (13–15). Recent single-cell transcriptomic study further revealed distinct ferroptosis-associated metabolic dysregulation in proximal tubular cells of IgAN patients (16). These results suggest that ferroptosis may represent a previously underappreciated mechanism contributing to renal injury and disease progression in IgAN. Therefore, it is important to conduct a systematic study of the expression of ferroptosis-related genes in IgAN, as it will help identify new molecular pathways linking ferroptosis to renal tissue damage and aid in the exploration of new potential therapeutic targets for IgAN.

**Figure 1:**
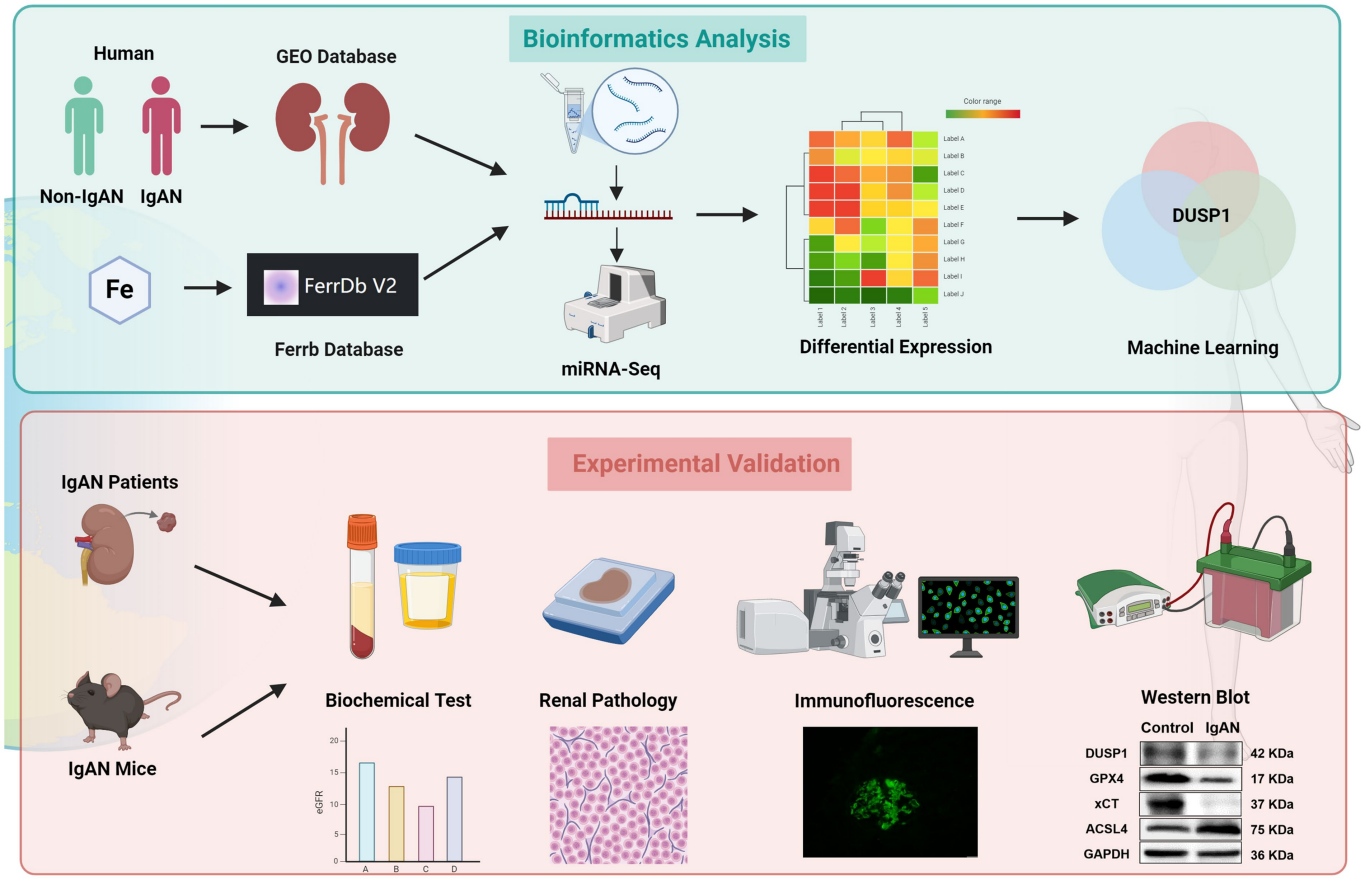
The flowchart of the study. Bioinformatics analysis: By integrating the clinical IgAN cohort, the GEO Database, miRNA-seq, and the Ferrb Database, through differential gene expression analysis and machine learning algorithms, DUSP1 was selected as the key target; Experimental Validation: Based on IgAN patients tissue and mouse models, through biochemical detection, pathological analysis, immunofluorescence, and western blot, multiple dimensions were used to verify the role of DUSP1 in the regulation of iron death in IgAN, following the logical chain of “omics screening → model validation,” to systematically present the analysis path of the iron death mechanism mediated by DUSP1.

This study utilized multiple machine learning approaches to identify ferroptosis-related feature gene (FFG) for the potential diagnosis of IgAN. While integrating clinical sample information for verification, we also constructed IgAN animal model for validations through traditional molecular biology experiments such as immunohistochemistry and western blot. Our findings demonstrated that dual-specificity phosphatase 1 (DUSP1) is downregulated in IgAN and significantly correlates with the expression levels of ferroptosis markers. To further elucidate the molecular interplay, Gene Ontology Set Enrichment Assessment (GSEA) was systematically conducted to delineate functional networks and pathophysiological processes implicating DUSP1 involvement in IgAN, and immune infiltration analysis was also carried out to elucidate its relationship with immune disorders. This discovery not only provides new insights into the molecular connection between IgAN and ferroptosis but also establishes a theoretical foundation for the development of targeted intervention strategies, highlighting the unique advantages of machine learning in unraveling complex disease mechanisms. The specific details of the study are depicted in Figure 1.

## 2 Materials and methods

### 2.1 Microarray data collection

We gathered three renal glomerular microarray datasets (GSE93798, GSE104948, GSE99339) associated with IgAN and normal control samples from the GEO database^1^ (17). Specifically, GSE93798 encompassed 20 IgAN and 22 control samples; and GSE104948 comprised 27 IgAN and 26 control samples. Both datasets were from the same microarray platform “GPL22945” and they were merged to form the training dataset, with batch effects adjusted through the SVA [version 3.50.0] package in R [version 4.3.1] (18). Furthermore, GSE99339 including 26 IgAN and 11 control samples was from another microarray platform “GPL19109” and served as the validation dataset, which could ensure the findings to be independent of the transcription platform selected. In cases where multiple probes corresponded to the same gene symbol, the average value was adopted as the representative gene expression level.

### 2.2 Identification of ferroptosis-related differential expressed genes

To identify the ferroptosis-related differential expressed genes (FDEGs) of IgAN, we first collected all the ferroptosis-related genes from Ferrb database, including ferroptosis drivers, inhibitors and markers. The Ferrb database^2^ is a manually updated database of ferroptosis (19). Then, expression data for the ferroptosis-related genes were obtained from the training dataset. The “limma” [version 3.56.2] R package was conducted to assess the differences in gene expression data (20). To control the proportion of false positives due to multiple testing, false discovery rate (FDR) correction was also applied. *FDR-p* value < 0.05 was used as the threshold to identify the significantly differentially expressed genes, which were identified as FDEGs.

### 2.3 Functional enrichment analysis

To get a deeper insight into the functional roles of the FDEGs, we conducted enrichment analyses for Gene Ontology (GO) and Kyoto Encyclopedia of Genes and Genomes (KEGG), while GO covers categories such as biological process (BP), cellular compartment (CC), and molecular function (MF) (21, 22). These analyses were performed using the “clusterProfiler” package [version 4.8.3] (23) with statistical significance defined by an adjusted *p* < 0.05. Data visualization was carried out via the following R packages: enrichplot [version 1.20.3] (23), circlize [version 0.4.16] (24), and ggplot2 [version 3.5.2] (25).

### 2.4 Filtering the ferroptosis-related feature gene

To screen the potential FFGs of IgAN, three algorithms were conducted to predict the disease status. They were least absolute shrinkage and selection operator, support vector machine-recursive feature elimination (SVM-RFE), and random forests (RF). LASSO is a regression analysis technique, utilizing regularization to enhance predictive accuracy. This method was applied via the “glmnet” [version 4.1.8] R package in this study, where the optimal model was determined by the minimum absolute shrinkage criterion (26). SVM is a widely used supervised learning approach for both regression and classification, often integrates the RFE algorithm to mitigate overfitting. SVM-RFE was carried out via the “e1071” [version 1.7.16] R package, employing fivefold cross-validation (27). RF is an ensemble technique that constructs multiple decision trees and importance scores greater than 2.0 was used to select genes for further analysis, which was performed using the “randomForest” [version 4.7.12] R package (28). Ultimately, the genes shared by the three algorithms were designated as the FFG.

### 2.5 Exploring the diagnostic value of FFG for IgAN

To evaluate the diagnostic performance of FFG, we constructed receiver operating characteristic (ROC) curves for mRNA expression data from training and validation datasets including renal tissue samples from patients with IgAN and normal controls. Diagnostic accuracy was then determined by calculating the area under the ROC curve (AUC) for each ROC. The ROC curves were generated via the “pROC” [version 1.18.5] R package (29), and a two-sided *p* < 0.05 was considered statistically significant.

### 2.6 Animals models

Eight-week-old male mice of the C57BL/6 J strain, which were used in the present investigation, were obtained from Beijing Weitonglihua Laboratory Animal Technology Co., Ltd. Standard animal chow and drinking water were provided by the Animal Experimental Center of the First Hospital of Jilin University. The rodents were housed under regulated environmental settings, specifically at a temperature of 22°C with a 12-h alternating light and darkness cycle. All experimental procedures involving these animals were executed in strict adherence to the guidelines sanctioned by the Animal Protection and Utilization Committee of the First Hospital of Jilin University. The rodents were assigned at random to either the control cohort or the IgAN cohort, comprising five animals in each group, as delineated in a prior publication (30, 31), an IgAN model was induced using bovine serum albumin, lipopolysaccharide (LPS), and carbon tetrachloride (CCL4). Particularly, the IgAN model group was subjected to a regimen involving BSA (administered intragastrically at a dose of 400 mg/kg, once every alternate day over a span of 6 weeks), CCL4 (administered via subcutaneous injection at a concentration of 100 μL dissolved in 300 μL of castor oil, once per week for 8 weeks), and LPS (administered via injection into the caudal vein at a dosage of 50 μg per animal at the 6th and 8th weeks). Following this, the mice were humanely euthanized, and samples of blood, urine, and kidney tissues were procured for subsequent analytical investigations.

### 2.7 Western blot

Fresh kidney tissue specimens were cleansed with pre-chilled phosphate-buffered saline (PBS) and subsequently subjected to homogenization in radioimmunoprecipitation assay buffer (NCM Biotech, China) fortified with 1% phenylmethanesulfonyl fluoride (PMSF) (Beyotime, China). The resulting lysate was then allowed to incubate at 4°C for a duration of 30 min, followed by centrifugation at 12,000 × g for 15 min under the same temperature conditions. The supernatant was thereafter harvested, and the total protein concentration was quantified utilizing a bicinchoninic acid (BCA) assay kit (NCM Biotech, China). The protein samples were standardized to a consistent concentration using lysate buffer, after which 5 × sodium dodecyl sulfate-polyacrylamide gel electrophoresis (SDS-PAGE) sample buffer (NCM Biotech, China) was introduced. The samples were then subjected to denaturation through boiling at 95°C for a 10-min duration. Protein separation was executed on a 10–15% gradient SDS-PAGE gel (ABclonal, China), with each well receiving a load of 30–50 μg of protein. The electrophoresis parameters encompassed a constant voltage of 80 V for 30 min in the stacking gel and 120 V for 60 min in the resolving gel. Proteins were transferred onto nitrocellulose membranes (NC membranes, Cytiva, United States) via the wet transfer method. To mitigate non-specific binding, the membranes were incubated with 5% skim milk powder at ambient temperature for a duration of 1 h. The following primary antibodies were sequentially incubated overnight at 4°C: anti-DUSP1 (1:1,000 dilution, 35217S, CST, United States), anti-GPX4 (1:5,000 dilution, ab125066, Abcam, United Kingdom), anti-xCT (1:1,000 dilution, ab307601, Abcam, United Kingdom), anti-ACSL4 (1:2,000 dilution, 22401-1-AP, Proteintech, China), and anti-GAPDH (1:1,000 dilution, 97166, CST, United States). On the subsequent day, horseradish peroxidase (HRP)-conjugated secondary antibodies (diluted at 1:10,000, Proteintech, China) were introduced and incubated at room temperature for a duration of 1 h. Protein bands were then visualized utilizing an ultra-sensitive ECL chemiluminescent substrate (Boster, China) and captured by means of a chemiluminescence imaging system (FUSION SoloS, Vilber, France). Image grayscale analysis was performed utilizing Image Lab 6.0 software (Bio-Rad), and the expression levels of the target proteins were normalized against GAPDH, which served as the internal reference standard.

### 2.8 Clinical sample collection

Renal Tissue Collection: Renal tissues from 51 patients diagnosed with IgAN based on renal biopsy pathology were procured from the First Hospital of Jilin University from May 2021 to May 2023. These patients were divided into 5 subgroups according to Lee’s pathological grading (8 with grade I-II, 12 with grade II, 15 with grade III, 10 with grade IV, and 6 with grade IV-V). Normal renal tissues were collected from 13 cases of solitary renal cell carcinoma adjacent to the kidney as the control group. Serum and Urine Sample Collection: Samples were collected from patients with IgAN confirmed pathologically via renal biopsy, as well as from healthy volunteers. The study received approval from the ethical committees (First Hospital of Jilin University, approval number 2023-508).

### 2.9 Renal histopathological examination and immunohistochemical staining

Renal tissue was preserved in 10% formalin for a duration of 24 h, subsequently embedded in paraffin, and sectioned into 5 μm-thick slices for pathological assessment and immunohistochemical staining. The kidney sections underwent deparaffinization and rehydration processes to evaluate alterations in their fundamental structure. Periodic acid-Schiff (PAS) staining was employed to assess glycogen content within the renal tissue, following previously established protocols. Periodic acid-Schiff methenamine silver staining was employed to examine the morphological characteristics of the glomerular mesangium and basement membrane. Assessment of renal fibrosis was carried out via Masson’s staining technique to visualize collagen deposition, as delineated in prior studies (32). Briefly, kidney sections were stained with the Sigma-Aldrich Trichrome Staining Kit for Masson’s staining. Immunohistochemical (IHC) staining with anti-DUSP1 (1:200 dilution, ab61201, Abcam, United Kingdom), anti-GPX4 (1:200 dilution, ab125066, Abcam, United Kingdom), anti-xCT (1:200 dilution, ab307601, Abcam, United Kingdom), anti-ACSL4 (1:300 dilution, 22401-1-AP, Proteintech, China) were also performed. All the stained sections were examined using an Olympus microscope VS200 system.

### 2.10 Renal immunofluorescence staining

Immunofluorescence staining procedures were executed following standard protocols outlined in prior research (33). Anti-IgA antibody (1:40 dilution, GF020421, Gene Tech, shanghai, China for patient; 1:200 dilution, 12-4,204-82, Invitrogen, United States for mouse) was applied for 30 min at the room temperature. The sections were overlaid with an aqueous mounting agent (Sigma-Aldrich) and examined under a fluorescence microscope (Olympus, Japan). Subsequently, all histological specimens were evaluated using the Olympus FV4000 confocal microscopy platform.

### 2.11 GSEA analysis for FFG

The IgAN patients in the training dataset were categorized into high and low expression groups, with the median expression level of the FFG gene serving as the cutoff. Enrichment pathways were identified through GSEA, using c5.go.symbols.gmt and c2.cp.kegg.symbols.gmt as gene set files (34). Pathways with a normalized enrichment score (NES) greater than 1 or less than −1, and a *p*-value < 0.05, were considered significantly enriched. Finally, the top six significantly enriched pathways were presented for visualization.

### 2.12 Immune cells infiltration analysis

The CIBERSORT algorithm was used to analyze the characteristics of immune cell infiltration in the renal tissue of the IgAN and control groups. CIBERSORT is a commonly utilized algorithm for analyzing immune cell infiltration. It employs linear support vector regression to break down the tissue expression matrix and accurately quantifies the relative abundance of 22 immune cell types in each sample (35). Principal component analysis of immune cell infiltration matrix was performed via the “ggplot2” R package. For visualization, “vioplot” [version 0.5.1] R package was used to draw the violin plot to directly represent the difference in immune cell infiltration between IgAN and controls. In addition, the “ggplot2” R package was utilized to further illustrate and visualize the Spearman correlation between immune infiltrating cells and the FFG.

### 2.13 Flow cytometry analysis

Mouse kidney tissues were collected and processed into a single-cell suspension. The cells were washed with pre-chilled phosphate-buffered saline (PBS) containing 2% fetal bovine serum (FBS), and then resuspended in staining buffer. Subsequently, fluorochrome-conjugated antibodies against CD3 (100203, BioLegend, United States) and CD4 (100543, BioLegend, United States) were added, and the cells were incubated in the dark at 4°C for 30 min. Following this staining step, the cells were washed again, resuspended in staining buffer, and analyzed using an Attune® Nxt acoustic focusing flow cytometer (Thermo Fisher Scientific). Data analysis was performed using FlowJo_v10.9.0 software. Cells were initially gated based on forward scatter (FSC) and side scatter (SSC) to exclude debris and doublets. CD3^+^ T cells and CD4^+^ T cell subsets were identified, and their infiltration ratios within the total cell population were calculated. The results are presented as the percentage of T cells relative to the total viable cell population.

### 2.14 Statistical analysis

Data were expressed as mean ± standard deviation (SD, *n* ≥ 5). Statistical evaluations were conducted using independent samples t-test for two groups and one-way analysis ANOVA with Tukey’s post-hoc test for multiple groups, utilizing GraphPad Prism 8.0.2 software (GraphPad Software, San Diego, CA). A *p*-value of < 0.05 was deemed statistically significant.

## 3 Results

### 3.1 Identification of ferroptosis-related differential expressed genes in IgAN

**Figure 2:**
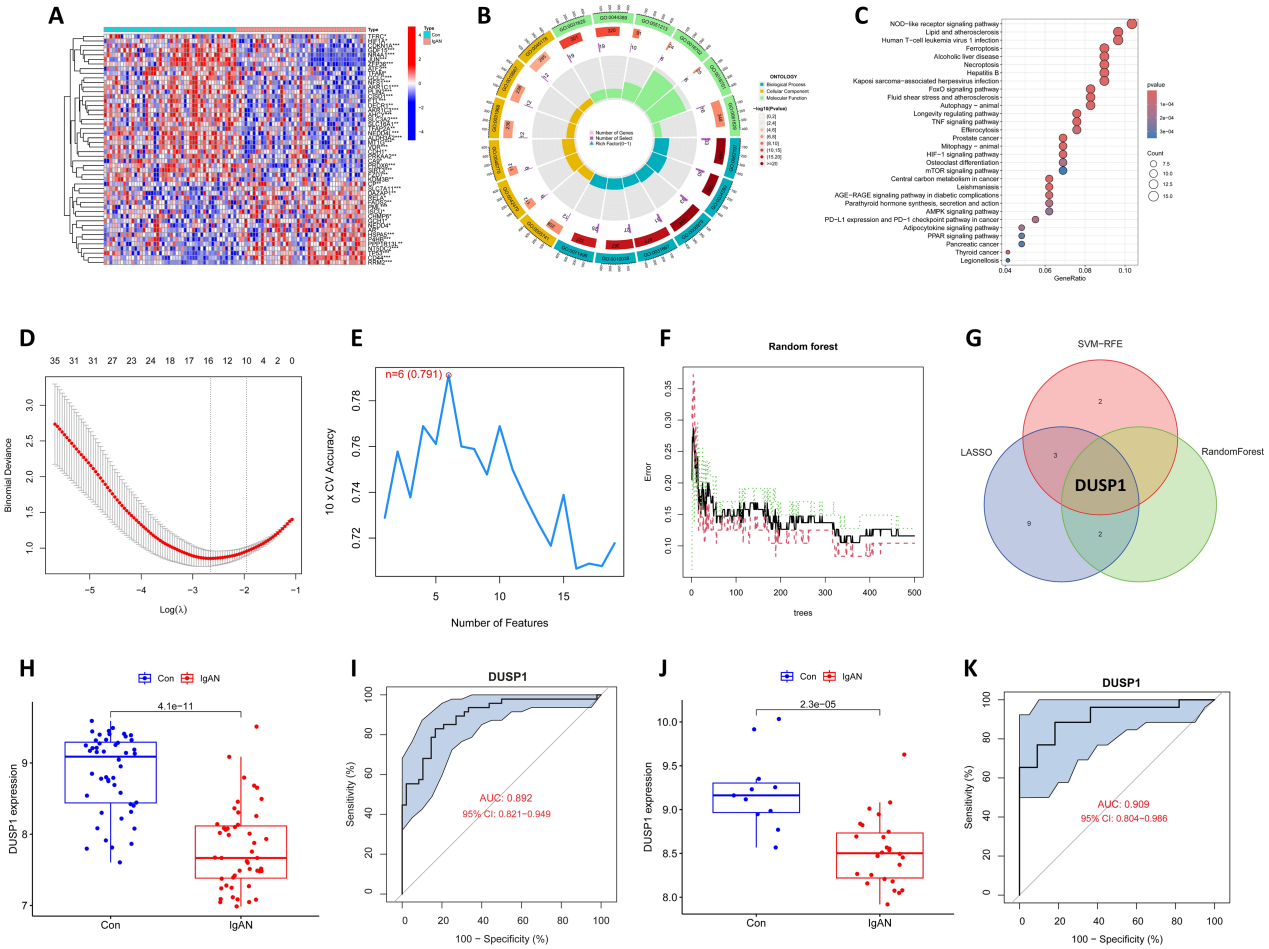
Bioinformatics analysis to identify ferroptosis-related feature gene (FFG) in IgAN. **(A)** Heatmap of the ferroptosis-related differential expressed genes (FDEGs) in IgAN. FDEGs highly expressed in the samples are marked in red, and FDEGs that are low in the samples are indicated in blue. **(B)** GO functional enrichment for the FDEGs. **(C)** KEGG pathway enrichment for the FDEGs. **(D)** Potential FFGs identified using LASSO logistic regression. **(E)** Potential FFGs identified using SVM-RFE. **(F)** Potential FFGs identified using RF algorithm. **(G)** Venn diagram of FFG identified using three algorithms. **(H)** The difference in expression of DUSP1 in training dataset. **(I)** ROC curve for DUSP1 using the training dataset, and the positive-class prevalence is 49.47% (*n*/positive = 47, *n*/negative = 48). **(J)** The difference in expression of DUSP1 in validation dataset. **(K)** ROC curve for DUSP1 using validation dataset, and the positive-class prevalence is 70.27% (*n*/positive = 26, *n*/negative = 11).

Renal microarray data from the training datasets of 47 IgAN samples and 48 controls obtained from two GEO data sets (GSE93798 and GSE104948) were retrospectively analyzed. The ferroptosis-related genes were obtained from the Ferrb database, and their expression data were extracted from the merged and corrected data. Differential analyses of the data were performed with statistical significance of *FDR-p* value <0.05, and a total of 180 FDEGs were identified, including 98 downregulated and 82 upregulated genes (Supplementary Table 1). The top 50 FDEGs with the most significant differential expression were presented in Figure 2A in the form of a heatmap, in which the clear separation between IgAN and control samples in the unsupervised clustering confirms the robustness of our normalization and batch correction procedures.

### 3.2 Functional enrichment analysis of FDEGs

Enrichment analyses for GO and KEGG were conducted on the identified FDEGs. The GO analysis revealed that the most significantly enriched terms in biological processes (BP) were cellular response to chemical stress, cellular response to oxidative stress and response to oxidative stress; in CC were mitochondrial outer membrane, melanosome and pigment granule; and in MF were ubiquitin protein ligase binding, ubiquitin-like protein ligase binding, and dioxygenase activity (Supplementary Table 2; Figure 2B). The strong enrichment of oxidative stress response terms suggests these FDEGs may mediate critical cellular defense mechanisms against oxidative damage, a process known to contribute to renal injury. Enrichment of MF with ubiquitin-related binding and dioxygenase activity, further support the potential roles of FDEGs in regulating protein homeostasis and oxygen-dependent metabolic processes, both of which are crucial for maintaining kidney cellular integrity. The KEGG enrichment analysis showed that the most significantly enriched pathways were the ferroptosis, Longevity regulating pathway, NOD-like receptor signaling pathway, FoxO signaling pathway and Necroptosis (Supplementary Table 3; Figure 2C). These pathways collectively highlight important connections to cell death regulation, stress resistance, and inflammatory responses—all key processes in renal pathophysiology.

### 3.3 Screening for ferroptosis-related feature genes

Three different algorithms demonstrating distinct but complementary selection patterns were conducted to identify the FFG of IgAN. LASSO logistic regression identified 15 genes reflecting its sensitivity to linear predictors (Figure 2D), SVM-RFE selected six genes highlighting the most margin-maximizing features (Figure 2E), and RF yielded three genes representing the most consistently important features across decision trees (Figure 2F). Notably, DUSP1 emerged as the only gene consistently selected by all three algorithms (Figure 2G), demonstrating consistent predictive power across linear, non-linear (SVM-RFE), and complex feature interactions (RF), and it was consequently classified as the FFG of IgAN.

### 3.4 Exploring the diagnostic value of FFG for IgAN

Box plot for the differential expression of DUSP1 in the training dataset was shown in Figure 2H, suggesting that DUSP1 was significantly downregulated in IgAN renal tissue. The ROC curve for DUSP1 in the training dataset was shown in Figure 2I, and its AUC was 0.892 (95% CI: 0.821–0.949). In addition, we further validated the differential expression and diagnostic efficacy of FFG in the validation dataset. As shown in Figure 2J, DUSP1 was also downregulated in IgAN renal tissue of validation dataset. Moreover, the AUC of ROC curve for DUSP1 in the validation dataset was 0.909 (95% CI: 0.804–0.986) (Figure 2K). In the ROC curves of both training and validation datasets, the high AUC value and stable confidence intervals of DUSP1 indicate that its differential expression in IgAN is robust and has a promising role to be a diagnostic biomarker for IgAN.

### 3.5 Expression of DUSP1 in clinical IgAN renal tissue and its relationship with pathological grades

**Figure 3:**
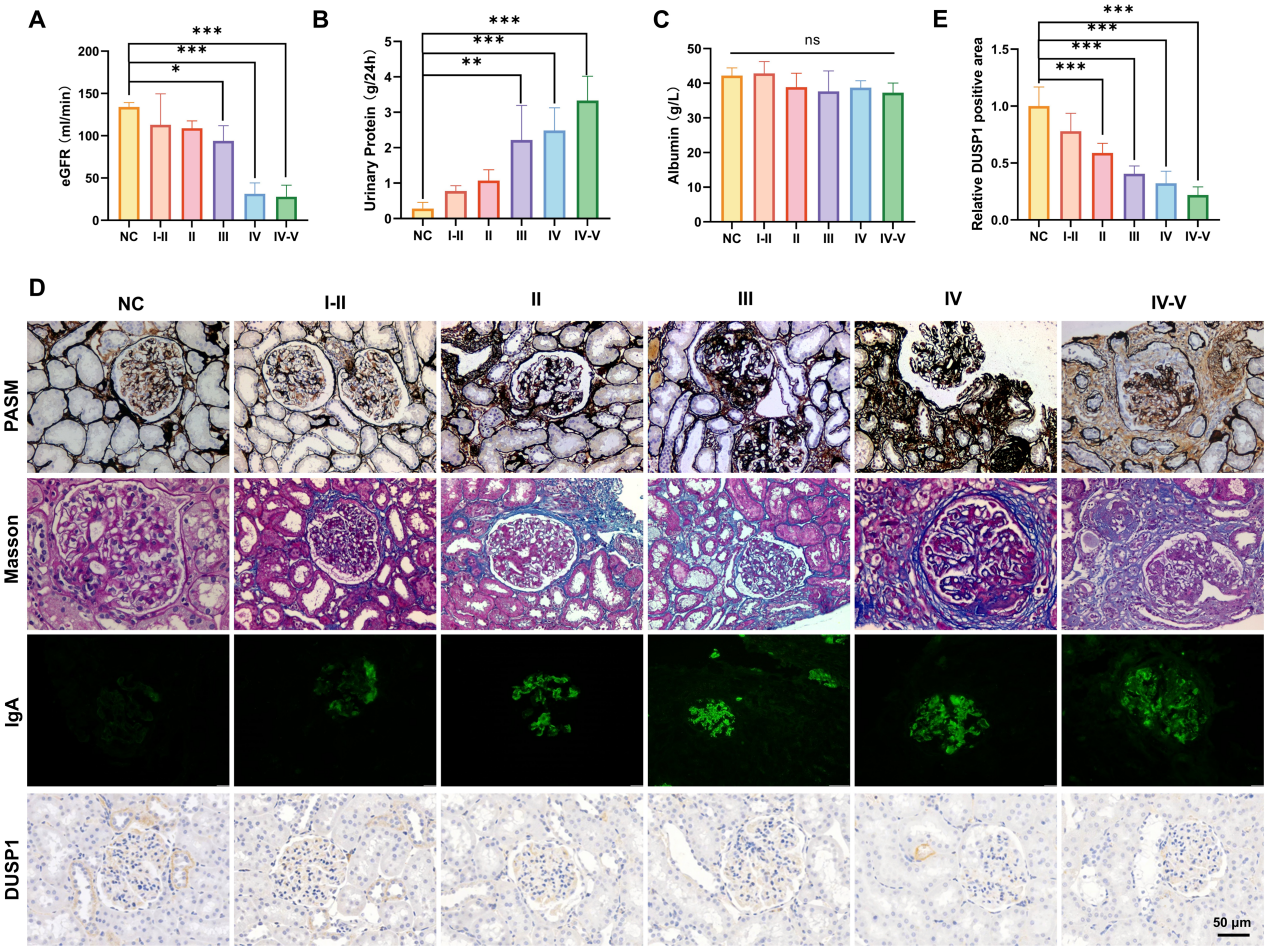
Physiological indicators, validation of identified genes DUSP1 and renal pathologic alterations in IgAN patients with different renal pathological grades. **(A)** Estimated glomerular filtration rate, **(B)** 24-h urinary protein quantification, and **(C)** serum albumin levels in patients. **(D)** PASM staining, Masson staining, IgA IF staining and DUSP1 IHC staining results of kidney tissues of patients. Relative positive area of DUSP1analysis show in **(E)**. Data were expressed as mean ± SD. Statistical evaluations were conducted via one-way ANOVA with Tukey post-hoc test. ^*^p < 0.05, ^**^p < 0.01, ^***^p < 0.001.

To systematically evaluate DUSP1 expression in IgAN and its potential clinical significance, we conducted a comprehensive analysis integrating renal function parameters, histopathological features, and DUSP1 expression patterns across different pathological grades. 51 patients with IgAN (8 with grade I-II, 12 with grade II, 15 with grade III, 10 with grade IV, and 6 with grade IV-V) and 13 control participants were included. Significant differences in physiological parameters were observed among IgAN patients stratified by renal pathological grades (Figures 3A–C). Patients with advanced pathological grades exhibited markedly reduced estimated glomerular filtration rates (eGFR, *p* < 0.05), elevated 24-h urinary protein excretion (*p* < 0.05), and decreased serum albumin levels. Histopathological analysis via PASM and Masson staining revealed progressive glomerular basement membrane thickening, mesangial matrix expansion, and interstitial fibrosis in higher pathological grades (Figure 3D). Immunofluorescence staining confirmed prominent IgA deposition in the mesangial regions, with intensity correlating positively with disease severity (Figure 3D). Immunohistochemical (IHC) analysis demonstrated significant downregulation of DUSP1 protein in renal tubular epithelial cells of IgAN patients compared to controls (*p* < 0.05), with expression levels inversely associated with pathological grading (Figures 3D,E). The findings suggest that DUSP1 may be a biomarker closely associated with IgAN progression, and its expression pattern is consistent with the pathological grading of the disease.

### 3.6 Investigation of the expression of DUSP1 and ferroptosis markers in the renal tissue of IgAN mice

**Figure 4:**
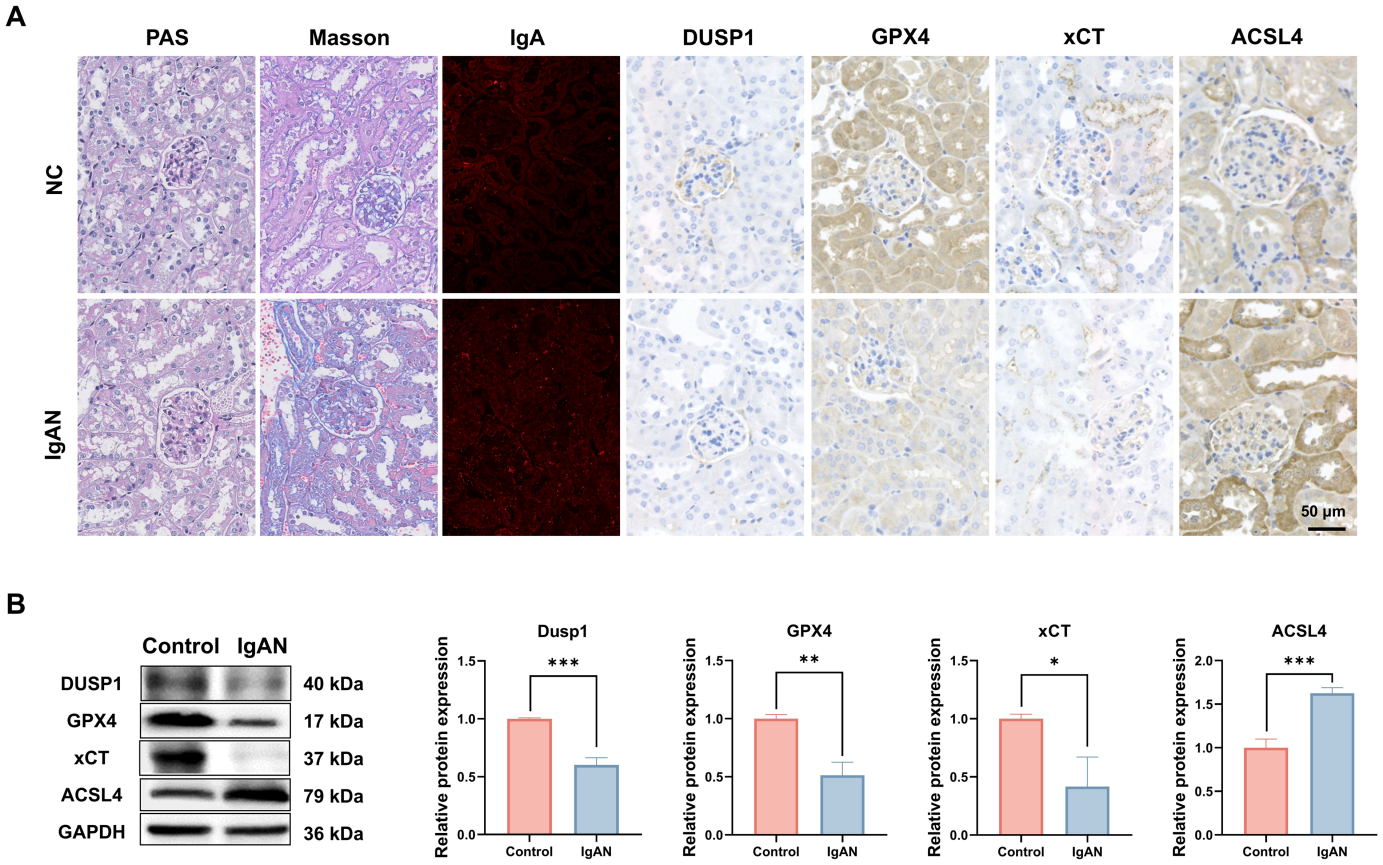
Investigation of the expression of DUSP1 and ferroptosis markers in the renal tissue of IgAN mice. **(A)** PAS staining, Masson staining, IF and IHC staining results of kidney tissues of control and IgAN mice. **(B)** Western Blotting for DUSP1, GPX4, xCT and ACSL4 proteins expression in Mice. Data were expressed as mean ± SD. Statistical evaluations were conducted using independent samples t-test. ^*^p < 0.05, ^**^p < 0.01, ^***^p < 0.001.

To further explore the role of ferroptosis in IgAN renal damage, we established an IgAN mice model via the mucosal immunization and investigated the expression of key ferroptosis markers and the identified FFG namely DUSP1 in renal tissues. As shown in Figure 4A, PAS and Masson staining in model mice highlighted glomerulosclerosis and interstitial fibrosis, and IF staining confirmed robust IgA deposition in glomeruli, recapitulating human IgAN features. IHC staining preliminarily showed that compared to normal controls, IgAN mice exhibited reduced DUSP1 expression in both glomerular and tubular areas, along with decreased expression of key ferroptosis markers xCT and GPX4, and increased expression of ferroptosis marker ACSL4. To quantitatively assess their expression levels, we also performed western blotting. As shown in Figure 4B, the results demonstrated that compared to control mice, the protein expression levels of DUSP1, xCT and GPX4 in kidney were significantly decreased (*p* < 0.05), while ACSL4 expression was significantly increased (*p* < 0.05). These findings further support the activation of ferroptosis in IgAN renal tissues and suggest that changes in DUSP1 expression may play an important role in this process.

### 3.7 Gene set enrichment analysis for FFG

**Figure 5:**
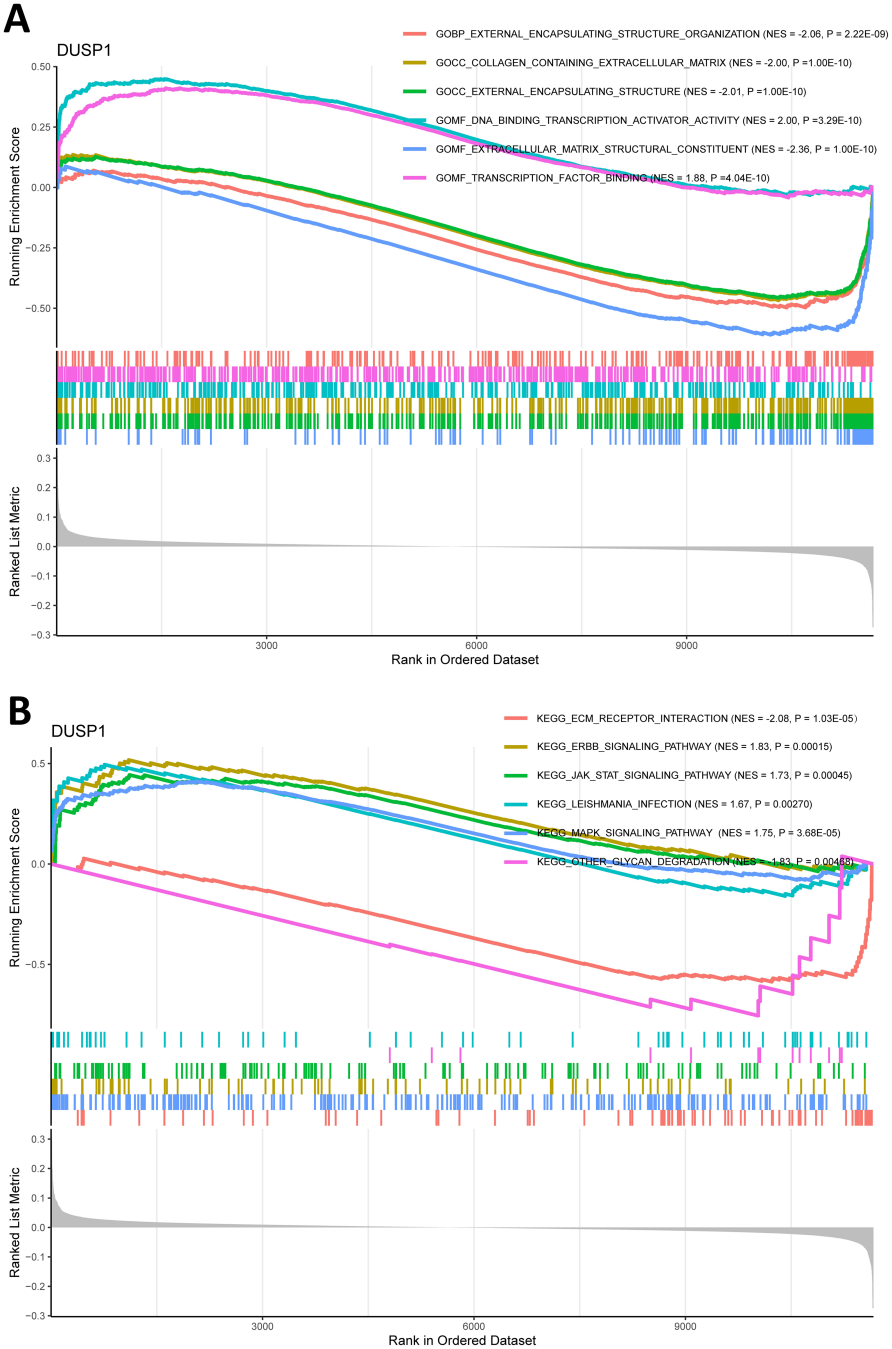
Gene set enrichment analysis (GSEA) for ferroptosis-related feature gene (FFG) in IgAN. **(A)** GO analysis of GSEA for DUSP1 in IgAN. **(B)** KEGG analysis of GSEA for DUSP1 in IgAN. Each pathway is labeled with its normalized enrichment score (NES) and *p*-value (weighted Kolmogorov–Smirnov test); |NES| > 1, and *p* < 0.05 are considered significant.

To explore the potential mechanisms of FFG in the development of IgAN, we split the training dataset IgAN samples into high expression and low expression two groups based on the median expression level of DUSP1 and performed GSEA analysis. GO analysis of GSEA found that DUSP1 was mainly involved in molecular functions such as extracellular matrix structural constituent, DNA binding transcription activator activity (Supplementary Table 4; Figure 5A). KEGG analysis using GSEA revealed that DUSP1 was primarily involved in several key signaling pathways, including the MAPK signaling pathway, ERBB signaling pathway, and JAK/STAT signaling pathway (Supplementary Table 5; Figure 5B). The difference in the number of enriched entries reflects both the disparate total sizes of the GO vs. KEGG libraries and the distinct underlying biology captured by each collection.

### 3.8 Immune cell infiltration and its correlation with FFG

**Figure 6:**
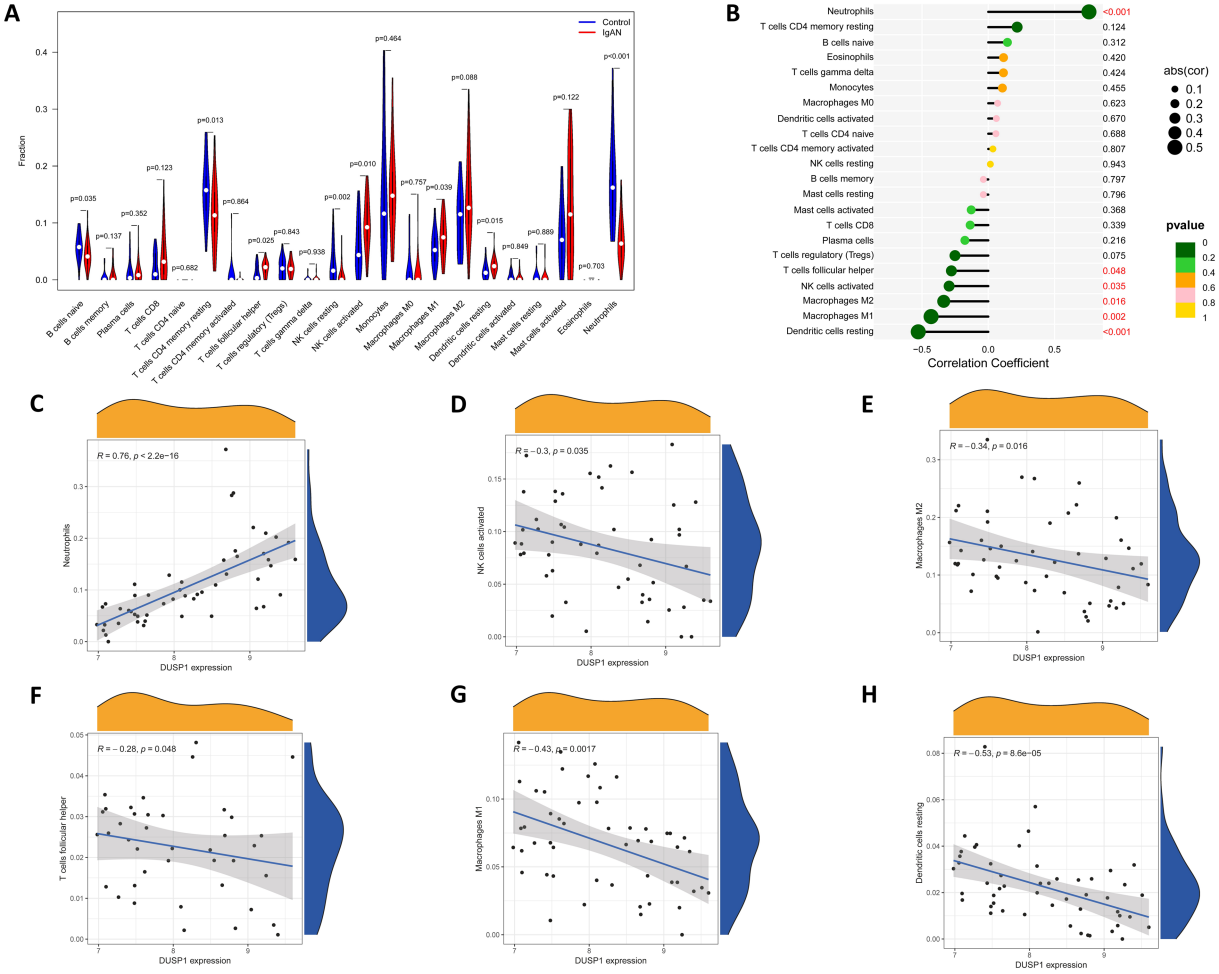
Immune cells infiltration analysis. **(A)** Violin plot of immune cells with differential infiltration between IgAN patients and controls based on CIBERSORT. **(B)** Overview of the correlations between DUSP1 expression and the extent of infiltration of immune cell subtypes. **(C)** Correlation between DUSP1 expression and Neutrophils. **(D)** Correlation between DUSP1 expression and NK cells activated. **(E)** Correlation between DUSP1 expression and Macrophages M2. **(F)** Correlation between DUSP1 expression and T cells follicular helper. **(G)** Correlation between DUSP1 expression and Macrophages M1. **(H)** Correlation between DUSP1 expression and dendritic cells resting. Differential immune cell infiltration between IgAN and control groups was assessed using the Wilcoxon rank-sum test, with a significance threshold of *p*-value < 0.05.

Since immune dysregulation plays an important role in the development of IgAN, we conducted an immune infiltration analysis to investigate the relationship between DUSP1 and immune cell infiltration in IgAN renal tissue, with the aim of further exploring its potential mechanisms involved in the development of IgAN. Firstly, we performed CIBERSORT analysis to investigate the characteristics of immune cell infiltration in renal tissues of IgAN compared to control group. As shown in Figure 6A, the violin plot of the differentially infiltrated immune cells found that T cells follicular helper, NK cells activated, Macrophages M1, Dendritic cells resting were present in larger numbers in IgAN samples than in control samples, whereas B cells naive, resting memory CD4^+^ T cells, NK cells resting, Neutrophils were present in smaller numbers in IgAN than control. Additionally, the correlation between the expression level of DUSP1 and the infiltrating immune cells in IgAN patients was also analyzed. The result revealed that the expression of DUSP1 exhibited correlations with immune cells. Specifically, DUSP1 displayed positive correlations with Neutrophils, and showed negative correlation with NK cells activated, Macrophages M1, Macrophages M2, T cells follicular helper and Dendritic cells resting. The details of the correlation analyses were displayed in Figures 6B–H.

### 3.9 Flow cytometry analysis of immune cell infiltration in renal tissues of IgAN mice

**Figure 7:**
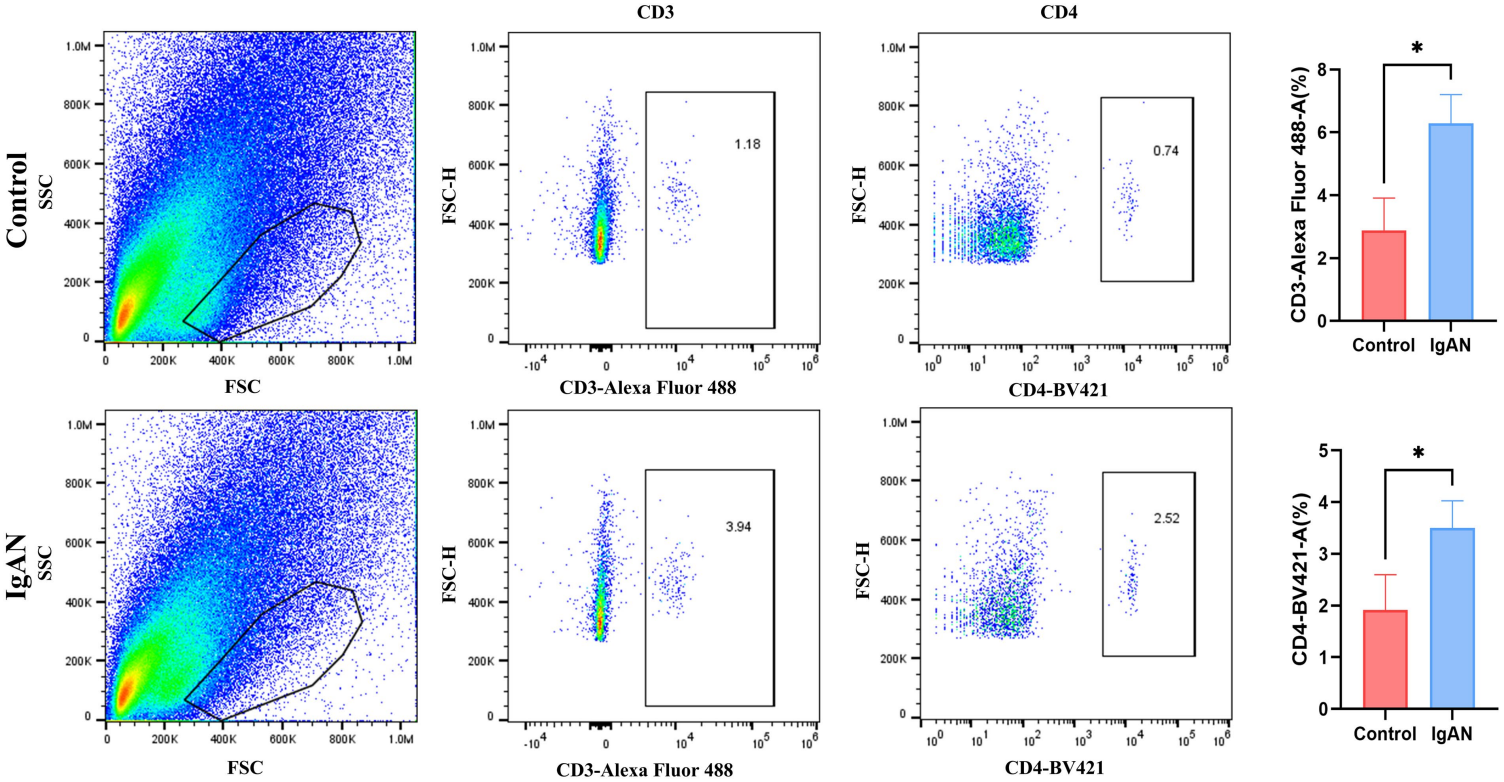
Flow cytometry analysis. Flow cytometry analysis of CD3^+^ and CD4^+^ T cell subsets in mouse kidney tissue. Data were expressed as mean ± SD. Statistical evaluations were conducted using independent samples t-test. ^*^p < 0.05.

To preliminarily validate the immune infiltration findings, flow cytometry analysis on renal tissues from both IgAN and control mice was conducted, and the results were shown in the Figure 7. T lymphocytes as key components of the immune system, primarily mediate cellular immune responses. Among the various T cell subsets, T follicular helper cells are characterized by the expression of CD3 and CD4 surface markers. Accordingly, this study employed anti-CD3 and anti-CD4 antibodies to assess T follicular helper cell infiltration in renal tissues. We compared the infiltration levels of CD3^+^ and CD4^+^ T cell subsets between the control group and the IgAN model group. The findings demonstrated that the proportions of CD3^+^ and CD4^+^ T cells in the renal tissues of the IgAN group were significantly elevated compared to the control group, with statistical significance (*p* < 0.05). These results align with the immune cell infiltration analysis derived from the CIBERSORT algorithm, further supporting the increased infiltration of T follicular helper cells in renal tissue during IgAN.

## 4 Discussion

Globally, IgAN stands as the most common variant of primary glomerulonephritis observed in adolescents. To date, symptomatic supportive treatment is still the main clinical treatment, encompassing angiotensin system blockers, sodium-glucose cotransporter 2 (SGLT2) inhibitors, and others. However, there is still a paucity of curative treatments targeting the core pathological mechanisms of the disease. Although novel targeted therapies, such as endothelin receptor antagonists and complement inhibitors, have been increasingly utilized in clinical practice in recent years, which can partially retard disease progression, the interplay between relapse susceptibility, inflammatory activity, and long-term prognosis following treatment remains unclear (36). The risk of patients progressing to ESRD remains substantial. Therefore, elucidating the pathogenesis of IgAN and identifying new therapeutic targets hold significant clinical implications. Initially proposed by Stockwell in 2012 (8), ferroptosis signifies a distinctive mode of programmed cell death that is propelled by lipid peroxidation, setting it apart from apoptosis, necrosis, and alternative cell death mechanisms. Emerging evidence indicates that ferroptosis plays a pivotal role in various kidney diseases. In acute kidney injury models, ferroptosis inhibitors significantly ameliorate renal dysfunction (37–40). In DN, activation of antioxidant pathways, such as Nrf2, has been shown to mitigate glomerulosclerosis by inhibiting ferroptosis (41, 42). In autosomal dominant polycystic kidney disease, ferroptosis promotes cyst growth, while its inhibition blocks this process (43). Additionally, ferroptosis exacerbates fibrosis via inducing oxidative damage in renal tubules and extracellular matrix (ECM) deposition (44). Downregulation of GPX4 expression and accumulation of lipid peroxides in lupus nephritis (LN) renal tissue further implicate ferroptosis in immune-mediated kidney injury (45, 46). Notably, recent studies suggest that ferroptosis may serve as a critical pathological trigger for IgAN. Dysregulated iron metabolism, imbalance in the antioxidant system, and the lipid peroxidation cascade may contribute to the inflammatory activation and fibrotic processes in IgAN through mediating glomerular intrinsic cell injury. However, systematic investigations into the direct association between ferroptosis and IgAN pathogenesis are lacking, particularly regarding specific molecular pathways, key regulatory targets, and potential intervention strategies.

DUSP1, also known as MAP Kinase Phosphatase-1 (MKP-1), belongs to a class of dual-specificity phosphatase enzymes. It negatively regulates key kinases in the MAPK signaling pathway, such as ERK, JNK, and p38, through dephosphorylation, thereby influencing cell proliferation, apoptosis, inflammation, and oxidative stress (47). In our study, the GSEA analysis via GSEA revealed that DUSP1 was mainly involved in several key signaling pathways, including the MAPK signaling pathway, the ERBB signaling pathway, and the JAK/STAT signaling pathway. Previous experimental studies have shown that DUSP1 has an improving effect on diabetic nephropathy (48). Sehoon et al. (49) found that after overexpressing DUSP1 in primary human renal tubular epithelial cells, it could improve inflammatory markers related to the MAPK pathway, suggesting that DUSP1 may be a potential strategy for treating tubulointerstitial injury. In addition, DUSP1 has been demonstrated to exert protective effects in various models of organ damage. For example, Xie et al. (50) reported that DUSP1 mitigates ischemia–reperfusion (I/R)-induced inflammatory responses and oxidative stress by suppressing excessive activation of the MAPK signaling pathway. Ferroptosis is characterized by the accumulation of iron-dependent lipid peroxides, and p38 as well as JNK within the MAPK signaling cascade have been implicated in promoting ferroptotic processes (51). As a negative regulator of the MAPK pathway, DUSP1 may inhibit ferroptosis by suppressing JNK/p38 phosphorylation, reducing ROS production, and mitigating lipid peroxidation (52). The Epidermal growth factor receptor (ERBB) tyrosine kinase family consists of four members, namely EGFR (ERBB1), HER2 (ERBB2), ERBB3 and ERBB4. Research has found that the expression of DUSP1 is controlled by downstream signals of EGFR and is downregulated in the presence of EGFR tyrosine kinase inhibitors. This down-regulation may be accompanied by the activation of JNK, thereby triggering the apoptotic pathway (53). Abnormal activation of the ERBB pathway has also been found in various cancers. For instance, overexpression of HER2 is an important driver of breast cancer, with ERBB2 amplification or overexpression present in approximately 20–30% of breast cancer patients. Studies have shown that DUSP1 is an important survival protein in breast cancer cells and a key downstream component of ERBB2 signaling (54). Accumulating evidence suggests that the JAK–STAT pathway contributes to renal tubulointerstitial fibrosis by mediating the actions of pro-fibrotic factors such as TGF-*β* during kidney injury (55). Therefore, it is hypothesized that DUSP1 may protect against renal oxidative stress and inflammation through dephosphorylation-mediated inhibition of these signaling pathways. Supporting this hypothesis, Lee et al. demonstrated that DUSP1 modulates macrophage-mediated inflammatory responses via regulation of STAT3 inhibition, thereby alleviating LPS-induced renal injury (56). The retrospective clinical analysis conducted in this study reveals that DUSP1 expression in renal tissues of patients with IgAN is significantly and negatively correlated with disease severity. Patients exhibiting low DUSP1 expression levels presented with higher 24-h urinary protein excretion, reduced eGFR, and more pronounced glomerular sclerosis and interstitial fibrosis on renal biopsy. The absence of DUSP1 expression may accelerate IgAN progression by enhancing the activity of the MAPK/NF-κB signaling pathway, which in turn promotes ferroptosis and extracellular matrix remodeling. These findings suggest that DUSP1 not only serves as a promising diagnostic biomarker for IgAN but also represents a potential therapeutic target through its modulation of ferroptotic and inflammatory processes.

Being a major glomerular disorder distinguished by the accumulation of immune complexes within the mesangial area, immunological imbalances are pivotal in the initiation and progression of IgAN. A growing body of research has revealed that the presence of immune cells in kidney tissue not only mirrors the intensity of renal disease activity but also underscores its underlying immune-mediated pathogenesis (9, 57). Therefore, in this study we utilized CIBERSORT analysis to explore the infiltration characteristics of different types of immune cells in the renal tissue of IgAN patients, with the aim of further understanding the pathogenesis of IgAN and helping to identify new biomarkers. Our analysis revealed a greater abundance of T follicular helper (Tfh) cells, activated NK cells, M1 macrophages, and resting dendritic cells in IgAN samples compared to control samples. Notably, Tfh cells are known to enhance B cell activation and antibody production during immune response (58). Therefore, they may be involved in the pathogenesis of IgAN by enhancing the production of Gd-IgA1 antibodies, which leads to an increase in the formation of circulating immune complexes. NK cells activated is closely associated with the initiation of immune response and may exacerbate local inflammatory responses (59). Macrophages M1 is also generally associated with pro-inflammatory effects (60). So, their infiltration may be involved in the damage and fibrosis of renal tissue. Additionally, we examined the association between DUSP1 expression and the infiltration of immune cells in IgAN kidney tissue. Our findings revealed an inverse correlation between DUSP1 levels and the presence of activated NK cells, M1 macrophages, M2 macrophages, and Tfh cells, among others. Combined with the fact that DUSP1 is significantly under-expressed in IgAN, this suggests that DUSP1 may play a regulatory role in the resting state of immune cells, possibly maintaining immune homeostasis by suppressing excessive immune responses. This finding provides new clues to understanding the role of DUSP1 in the immune response in IgAN and suggests that DUSP1 may be a potential intervention target for future immunomodulatory therapies.

In summary, our study systematically elucidated the potential regulatory role of DUSP1 in the pathogenesis of IgAN through integrated bioinformatics analyses, retrospective clinical cohort validation, and molecular phenotyping in a murine IgAN model. Our findings revealed significant downregulation of DUSP1 expression in renal tissues from both IgAN patients and model mice, with its expression levels closely correlated with ferroptosis markers, thereby providing novel insights into the mechanisms underlying IgAN pathogenesis. Notably, future investigations could further extend these findings in the following directions: (1) Comprehensive functional validation using conditional knockout or overexpression models is required to delineate the precise molecular pathways through which DUSP1 modulates IgAN progression, thereby uncovering its multidimensional regulatory mechanisms; (2) Establishing longitudinal cohorts with dynamic monitoring of DUSP1 expression profiles may enhance its clinical prognostic significance by elucidating temporal correlations with disease progression and therapeutic outcomes; (3) Multi-omics approaches incorporating spatiotemporal resolution are warranted to dissect downstream signaling hubs along the DUSP1-ferroptosis axis and their interplay with hallmark IgAN pathological features, such as mesangial cell proliferation and complement deposition. Collectively, these proposed investigations will refine the DUSP1 regulatory network and provide a foundation for targeted therapeutic strategies in IgAN.

## Data Availability

The chip data analyzed in this study were obtained from Gene Expression Omnibus (GEO) database (http://www.ncbi.nlm.nih.gov/geo/). GSE93798 was downloaded from https://www.ncbi.nlm.nih.gov/geo/query/acc.cgi?acc=GSE93798, GSE104948 was downloaded from https://www.ncbi.nlm.nih.gov/geo/query/acc.cgi?acc=GSE104948, and GSE99339 was downloaded from https://www.ncbi.nlm.nih.gov/geo/query/acc.cgi?acc=GSE99339.

## Footnotes

^1^ https://www.ncbi.nlm.nih.gov/geo/

^2^ https://www.zhounan.org/ferrdb/current
