# Supplementary material for: Bioinformatic analysis, clinical implications and experimental validation of ferroptosis-related feature gene in IgA nephropathy: focus on DUSP1
Source: Front Med (Lausanne). 2025 Aug 7;12:1612200. doi: 10.3389/fmed.2025.1612200 (PMC12367746; doi:10.3389/fmed.2025.1612200)
Supplement: Supplementary file 1 [file Table_1.docx]

Supplementary Material

# Supplementary Tables

Supplementary table 1: The information of ferroptosis-related differential expressed genes

Supplementary table 2: The information of the GO enrichment analyses of the identified FDEGs

Supplementary table 3: The information of the KEGG enrichment analyses of the identified FDEGs

Supplementary table 4: The information of the GSEA-GO enrichment analyses of the DUSP1

Supplementary table 5: The information of the GSEA-KEGG enrichment analyses of the DUSP1

**
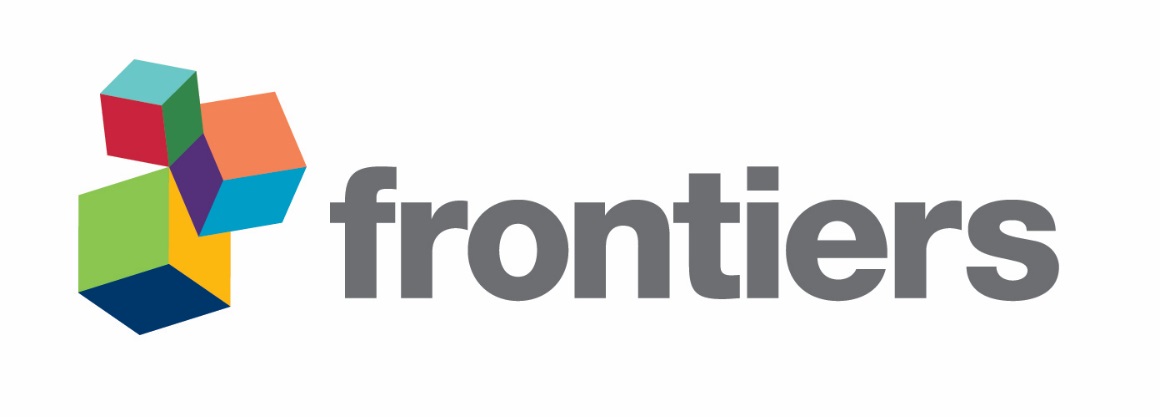
**
